# Supplementary material for: The genome sequence of the solitary wasp, Nysson trimaculatus (Rossi, 1790) (Hymenoptera: Crabronidae)
Source: Wellcome Open Res. 2026 May 2;11:257. [Version 1] doi: 10.12688/wellcomeopenres.26493.1 (PMC13227096; doi:10.12688/wellcomeopenres.26493.1)
Supplement: Peer review history

# Reviewer report: Reviewer response for version 1

**DOI:** 10.21956/wellcomeopenres.29174.r155343

**Published:** 2026-06-05

**Authors:** Din Syafruddin

**Affiliations:** 1 Universitas Hasanuddin, Makassar, Indonesia

The manuscript is well written and presented, except for detail chromosomal content (for the chromosomal genome), and gene content for the mitochondrial genome. (it may be accessible through supplementary files, but would probably be informative to summarize it in the abstract. Arrangement of the chromosomes and mitochondrial genome should also be presented as map in graphical abstract

Are sufficient details of methods and materials provided to allow replication by others?

Yes

Is the rationale for creating the dataset(s) clearly described?

Yes

Are the datasets clearly presented in a useable and accessible format?

Partly

Are the protocols appropriate and is the work technically sound?

Yes

Reviewer Expertise:

Molecular parasitology and entomology

I confirm that I have read this submission and believe that I have an appropriate level of expertise to confirm that it is of an acceptable scientific standard, however I have significant reservations, as outlined above.

# Reviewer report: Reviewer response for version 1

**DOI:** 10.21956/wellcomeopenres.29174.r155344

**Published:** 2026-06-03

**Authors:** Marta Coronado-Zamora, Vicente Chillida

**Affiliations:** 1 Departament de Genetica i de Microbiologia, Universitat Autònoma de Barcelona - UAB Barcelona, Bellaterra, Catalonia, Spain; 2 Autonomous University of Barcelona Department of Genetics and Microbiology, Bellaterra, Catalonia, Spain

This data note describes a high quality chromosome assembly for the solitary wasp ( *Nysson trimaculatus*). The genome data are available at NCBI BioProject, and data is accessible as checked in May 2026. The genome assembly is highly contiguous, with overall very good assembly metrics. The mitochondrial genome is also included. 

 Comments:

 - The genome currently lacks gene annotations, although it is stated that it will be annotated with available RNA-seq. However, it is not clear if the RNA-seq was produced within this study or it was already available. 

 - Regarding haplotype 2, why was it assembled to the scaffold level while haplotype 1 was able to be assembled to the chromosome level? 

 - fasta_windows is mentioned in table 5 but not in the main text 

 - As DNA concentration was measured with Qubit and Nanodrop, it would be nice for the authors to report 260/280 and 260/230 ratios for DNA purity from Nanodrop

 Typos:

 - Extra white space in  “( Figure 2).“

Are sufficient details of methods and materials provided to allow replication by others?

Yes

Is the rationale for creating the dataset(s) clearly described?

Yes

Are the datasets clearly presented in a useable and accessible format?

Yes

Are the protocols appropriate and is the work technically sound?

Yes

Reviewer Expertise:

Population genomics, bioinformatics, transposable elements

We confirm that we have read this submission and believe that we have an appropriate level of expertise to confirm that it is of an acceptable scientific standard.

# Reviewer report: Reviewer response for version 1

**DOI:** 10.21956/wellcomeopenres.29174.r155345

**Published:** 2026-06-01

**Authors:** Sharu Paul Sharma

**Affiliations:** 1 Iowa State University, Iowa, USA

This manuscript presents a high-quality genome assembly for the solitary wasp (Nysson trimaculatus), generated as part of the Darwin Tree of Life project. The study is clearly written and provides a useful genomic resource for future work.

 A 25.2 kb mitochondrial assembly is longer than typical insect mitochondrial genome, so a brief note on completeness, duplicated/control-region content, or circularization would be useful.

 Overall, the manuscript is clear and concise with metrics expected of a high quality assembly including high BUSCO score.

Are sufficient details of methods and materials provided to allow replication by others?

Yes

Is the rationale for creating the dataset(s) clearly described?

Yes

Are the datasets clearly presented in a useable and accessible format?

Yes

Are the protocols appropriate and is the work technically sound?

Yes

Reviewer Expertise:

Bioinformatics

I confirm that I have read this submission and believe that I have an appropriate level of expertise to confirm that it is of an acceptable scientific standard.

# Reviewer report: Reviewer response for version 1

**DOI:** 10.21956/wellcomeopenres.29174.r155341

**Published:** 2026-05-22

**Authors:** Vahap Eldem

**Affiliations:** 1 Istanbul University, Istanbul, Turkey

The manuscript presents a chromosome level genome assembly for the solitary wasp Nysson trimaculatus, generated using PacBio HiFi and Hi-C data. Overall, the assembly appears to be of high quality, with high BUSCO completeness and strong consensus accuracy.

 The manuscript would benefit from clarifying a minor taxonomic inconsistency. The title, NCBI taxonomy, and species taxonomy section appear to place N. trimaculatus in Crabronidae, whereas the background text states that the species belongs to Bembicidae. This should be clarified.

 Overall, this is a valuable genome data note and appears suitable for indexing after minor clarification.

Are sufficient details of methods and materials provided to allow replication by others?

Yes

Is the rationale for creating the dataset(s) clearly described?

Yes

Are the datasets clearly presented in a useable and accessible format?

Yes

Are the protocols appropriate and is the work technically sound?

Yes

Reviewer Expertise:

Genomics, Bioimformatics

I confirm that I have read this submission and believe that I have an appropriate level of expertise to confirm that it is of an acceptable scientific standard.
